# Genome editing for improving nutritional quality, post-harvest shelf life and stress tolerance of fruits, vegetables, and ornamentals

**DOI:** 10.3389/fgeed.2023.1094965

**Published:** 2023-02-24

**Authors:** Punam Sharma, Anuradha Pandey, Rinku Malviya, Sharmistha Dey, Subhasis Karmakar, Dipak Gayen

**Affiliations:** ^1^ Department of Biochemistry, School of Life Sciences, Central University of Rajasthan, Ajmer, India; ^2^ ICAR-National Rice Research Institute, Cuttack, India

**Keywords:** CRISPR/Cas9, postharvest loss, fruits, vegetables, gene editing

## Abstract

Agricultural production relies on horticultural crops, including vegetables, fruits, and ornamental plants, which sustain human life. With an alarming increase in human population and the consequential need for more food, it has become necessary for increased production to maintain food security. Conventional breeding has subsidized the development of improved verities but to enhance crop production, new breeding techniques need to be acquired. CRISPR-Cas9 system is a unique and powerful genome manipulation tool that can change the DNA in a precise way. Based on the bacterial adaptive immune system, this technique uses an endonuclease that creates double-stranded breaks (DSBs) at the target loci under the guidance of a single guide RNA. These DSBs can be repaired by a cellular repair mechanism that installs small insertion and deletion (indels) at the cut sites. When equated to alternate editing tools like ZFN, TALENs, and meganucleases, CRISPR- The cas-based editing tool has quickly gained fast-forward for its simplicity, ease to use, and low off-target effect. In numerous horticultural and industrial crops, the CRISPR technology has been successfully used to enhance stress tolerance, self-life, nutritional improvements, flavor, and metabolites. The CRISPR-based tool is the most appropriate one with the prospective goal of generating non-transgenic yields and avoiding the regulatory hurdles to release the modified crops into the market. Although several challenges for editing horticultural, industrial, and ornamental crops remain, this new novel nuclease, with its crop-specific application, makes it a dynamic tool for crop improvement.

## 1 Introduction

In the present world, chronic malnourishment engulfs at least one billion population with the loss of biodiversity, climate change, and continuously degrading agriculture systems (Foley et al., 2011). Contemporary agriculture systems will face an enormous problem as the world population increases day by day with an estimated 9 billion by 2050 (Gomiero et al., 2011). The yield enhancement spawned during the green revolution is now steady diwindling because of rapid climate change which limits crop production and needs plants that can resist adverse environments with a higher yield. Conventional breeding is a dilatory and laborious process, a more efficient and systematized method is much required (Ashkani et al., 2015). With the availability of genomic sequences for multifarious plants, genome editing furnishes with an opportunity to edit crop varieties with greater precision. This strategy includes sequence-specific nucleases which can induce DNA double-stranded break (DSB) at the specific target sites. Following DSBs, repairs are made through donor template-dependent homology dependent repair (HDR) or error-prone non-homologous end joining pathways that introduce tiny insertion and deletion (indels) events at the target sites (Schmidt et al., 2019).

CRISPR (Clustered regularly interspaced short palindromic repeat) -Cas (CRISPR-associated protein) is an adaptive immune system discovered in bacteria and archaea, relying on RNA-DNA interaction and nuclease for targeted cleavage. With minimal effort and cost, DSBs may be readily induced at any desired target genomic locations using CRISPR-Cas and their orthologs (Kumlehn et al., 2018).

Genome editing technology has better efficiency to change the genome architecture at precise locations, with appropriate accuracy. The generation of new varieties of plants producing fecund yields with environmental stress resistance has been possible using this genome editing approach. It is more difficult to modify all of the plant genes using a specific genome editing technology because of the complicated architecture of the plants. However, several genome editing technologies have been created that have improved genome editing in plants to overcome this type of problem. Some of the genome editing technology employed to edit genomes of the plant is CRISPR/Cas9, ZFNs (zinc finger nucleases), HR (homologous recombination), TALENs (transcription activator-like effector nucleases). Additionally, oligonucleotide-directed mutagenesis and editing of the site-directed sequence have the efficiency of genome editing at the single-nucleotide level. Lately, the ABEs (adenine base editors) has been developed to mutate A-T base pairs to G-C base pairs. The field of genome-based breeding has gained new opportunities because of genome editing technology. Earlier genome editing tools such as meganuclease (Silva et al., 2011), zinc finger nucleases (ZFNs) (Carroll, 2011), and transcription activator-like effector nucleases (TALENs) (Sun and Zhao, 2013) have shown effectiveness for PGE (plant genome editing), but require complex protein engineering for each target site. CRISPR-Cas has been used for a large number of crops to modify traits with great economic value (Li et al., 2020). CRISPR-Cas mediated genome editting offers fewer risks as compared to GM crops as the majority of edits involve few nucleotides change (Ahmad et al., 2021). As the editing reagents are segregated out in the subsequent generation there is no distinction between a natural mutant and a mutant caused by genome edit. Thus, the incorporation of genome editing in normal breeding practices should enhance the speed of precision crop breeding and improve modern agriculture (Miki et al., 2018). Fruits and vegetables are more nutrient-dense and include more bioactive phytochemicals, which are essential for the global population (Alfa and Arroo, 2019; Fraga et al., 2019; Liskova et al., 2019; Saiwal et al., 2019). Plants experience several environmental factors like dehydration, heat, cold, pathogen, and many other harmful abiotic and biotic stress. Around 25 to 40 percent of the fruits and vegetables grown worldwide are never consumed after being harvested (Gustavsson et al., 2011). Fruit, vegetable, and ornamental plant losses are estimated to be around 75% (Kitinoja and Tokala, 2018; Porat et al., 2018). Production of fruit, vegetable, and ornamental plant in developing countries is already insufficient to meet people’s nutritional needs (Kc et al., 2018). Worldwide efforts have been initiated to reduce the anticipated loss of fruits, vegetables, and ornamental plants. However, the causes of postharvest waste and loss are very complex. Technology-based breeding for new and improved fruits, vegetables, and ornamental plants provides better quality which is a crucial component of the long-term solution. The most significant advancement in plant breeding since the green revolution is gene editing. It has already been used to uncover new information about plants, and it also holds enormous promise for creating new crops with various desired traits (Wolter et al., 2019). Genome editing technology is used in plants to produce novel phenotypes that increases agricultural productivity.

In several commercial crops, successful application of the CRISPR-based genome editing tools have been utilized. It is for improving resistance against diverse biotic and abiotic stresses in association with enhanced quality of fruits and grain architectures (Parmar et al., 2017). In this comprehensive study, we explain the potential use of genome editing tools that can create a robust and measurable platform to improve traits related to nutritional quality, post-harvest yield losses, and resistance against biotic and abiotic stresses. The gene and genome editing tools viz, ZFN, TALEN, CRISPR/Cas9, and also base editing, prime editing, and epigenome editing tools has been discussed in a concise way. Finally, we discuss gene editing technologies and improvement strategies, which will provide a resource for new researchers for crop improvement.

## 2 Tools for genome editing

A slight modification or epigenetic change in the genome structure or gene structure of any organism can tune up or bring about desired changes, such as better yield, high tolerance, and high storage susceptibility. Modern technology has allowed scientists to perform controlled alterations in the gene structure within plant genomes which can be widely classified as Gene Editing or Gene targeting. Any technique which can perform permanent modification at a specific site is called genome targeting and is widely used for the improvement of the plant. The cellular DNA repair mechanisms are triggered when a Double-Stranded Break (DSBs) occurs. Either the homology-directed repair (HDR) mechanism or the non-homologous end joining (NHEJ) technique mechanism repairs the double-stranded breaks. The first one being sensitive to error, causes many additions or deletions, thus causing deterioration of the gene or its by-product. The Second one although error-prone yet causes small changes like addition of transgene. This allows for the targeted mutagenesis that results from engineering the target locus.

In plant species, the NHEJ repair mechanism is more prevalent than HDR because the latter depends on both structure of donor DNA and the repair machinery of the target cell. It has proven to be a tough assignment; hence NHEJ is chosen as a better and more relevant approach. The discovery of innovative specific and rare-cutting restriction enzymes that can induce double-stranded breaks at specific genomic sites is essential for the development of DSB-dependent Genome Editing technologies in plant cells. Nucleases recognize long nucleotide sequences and bring upon double-stranded breaks at their target sites. These meganucleases can be engineered as per the target sequence to be mutated. The gene editing technologies uses different engineered nucleases such as meganucleases, zinc finger nucleases (ZFNs), Transcription activator-like effector nucleases (TALENs), and newly added CRISPR-Cas endonuclease (Mao et al., 2019). All these designed nucleases share the trait of coupling a programmable DNA binding activity with a sequence-independent endonuclease activity that is sufficiently selective to target a particular genomic location.

### 2.1 Nuclease-mediated gene editing technologies

#### 2.1.1 Zinc finger nucleases

**FIGURE 1:**
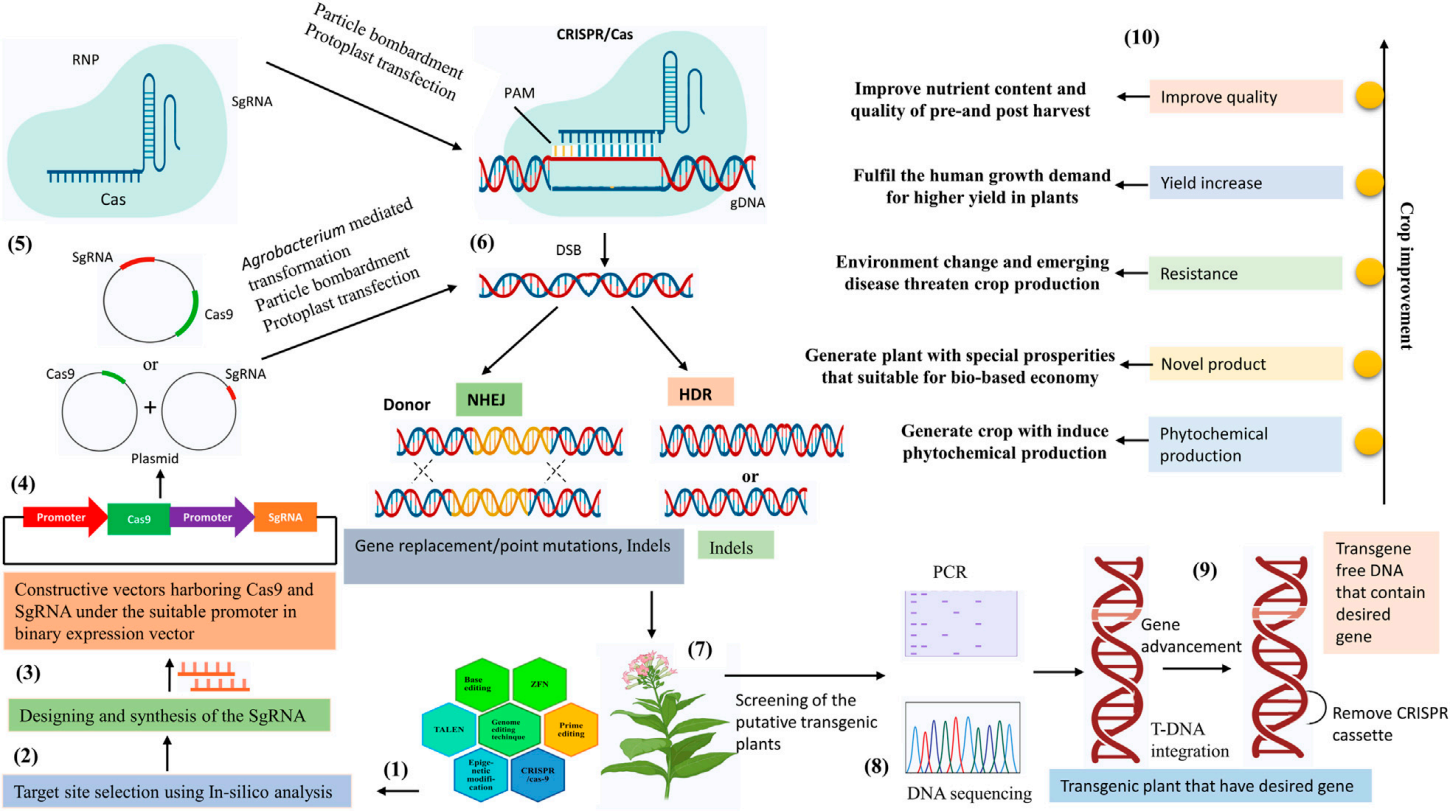
Overview of the CRISPR/Cas9 mediated genome editing in the plant. (1) Genome editing techniques. (2) Selection of the genomic target using In-silico analysis. (3) Design the sgRNA complementary to the target sequence. (4) Cloning of the designed Cas9 and sgRNA under suitable promoter in the binary expression vector. (5) The components of CRISPR/Cas9 tools construct transfer into plant cells, *via Agrobacterium-*mediated transformation, protoplast transfection, and particle bombardment. (6) CRISPR/Cas9 tools mediated genome editing in plants depend on the two main DSB (double-strand break) pathways. Gene replacement/point mutations, and indels are outcomes of the NHEJ (non-homologous end joining) pathway. Gene indels are outcomes of the HDR (homology-directed repair) pathway. (7) Development of the transgenic plant. (8) Identify the mutations in transgenic plants using DNA sequencing and PCR restriction enzyme assays and genotyping the transgenic plant with the desired mutation. (9) Removal of the CRISPR/Cas9 cassette (10) Crop improvement by the CRISPR/cas9 technology.

ZFN is an artificially engineered endonuclease system consisting of Zinc Finger Protein (ZFP) fused with the *Fok*1 restriction enzyme. Zinc Finger Protein consists of approximately 30 conserved amino acids in ββα configuration, which are classified as Type II endonuclease (Gaj et al., 2013). ZFN comprised two domains responsible for DNA cleavage; a synthetically designed Cys-Cys-His-His Zinc finger domain at the N-terminal and the non-specific DNA cleavage Domain of the *Fok*1 endonuclease at the C-terminal. ZFN domain has three to four zinc fingers, each of which can recognize three base pairs long stretch of the target DNA sequence and hence allows proper tethering with the target DNA (Petolino, 2015). The *Fok*1 cleavage domain dimerization is essential to cut the target DNA sequence. Two ZFN monomers orient themselves in opposite directions to the target DNA site (Figure 1). They are designed such that there is a flank of 5-6 bps DNA sequence between the two monomers, which becomes the restriction site of dimerized *Fok*1 endonuclease (Weinthal et al., 2010). Once the target site is cut, the cellular repair mechanism of the Non-Homologous End Joining mechanism repairs it with some errors like Insertions and deletions. ZFNs have been employed in model and crop plant species as site-specific mutagens to facilitate the integration of the targeted transgene into donor genome sequences, to promote the repair of defective transgenes, to replace donor DNA sequences with foreign DNA molecules, and to ease the integration of the targeted transgene into donor genome sequences.

#### 2.1.2 Transcription activator like effector nucleases (TALENs)

TALENs were developed as the fusion of Transcription Activator Like Effector with the catalytic domain of *Fok*1 endonuclease. TALEs proteins are found naturally in *Xanthomonas proteobacterium* and are involved in plant infection (Malzahn et al., 2017). During infection, they transfer the TALE proteins to the plant’s cell’s nucleus, bind to the promoter region of the target gene, and activate transcription (Figure 1). The structure of TALENs consists of three different domains: the N terminal contains a type III secretion system (T3SS), non-canonical repeats (NCR), and the C terminal has a transcription factor binding site, Nuclear Localization Signal (NLS), and an activation domain (Becker and Boch, 2021). Between these two domains, there exists a stretch of conserved 34 amino-acid long tandem repeats. Among them, the amino acid at the 12th and 13th positions are the ones responsible for the specificity of the TALENs. As in ZFN, TALENs also function as dimers, operating in opposite directions. When these two opposite TALENs fuse on the target gene, their Fok1 domain is responsible for mediating double-strand break. With the cell’s repair property, the DSB is repaired either by NHEJ or HDR mechanism resulting in insertion, deletion, or substitution and forming a mutant or knock-out variety.

#### 2.1.3 CRISPR/cas system

CRISPR/Cas system was found to naturally exist as an RNA-guided DNA endonuclease system in the prokaryotic organism mostly by archaea and eubacteria. It helped to develop a target-specific immune response against foreign invaders, primarily viruses and plasmids, with the aid of the Cas protein, which cleaves the target sequence specifically and creates DSBs in the target DNA of the invading organism and hence destroys them. CRISPR stands for Clustered Regularly Interspaced Short Palindromic Repeats, and Cas are the CRISPR-associated genes. CRISPR consists of highly conserved short tandem repeats separated by short spacer sequences. Spacer sequences are unique sequences homologous with exogenous viral or plasmid DNA sequences (Bhowmik et al., 2021). These sequences are further used as recognition elements of foreign viral or plasmids and help bacteria to eliminate them. This is defined as the defense mechanism adopted by archaea and eubacteria. This technology is widely accepted and recognized for serving as machinery for genome editing and gene therapy for any organism/living system. It is the most precise, accurate, simple, and site-specific method of genetic engineering in plant systems. This technology has an immense capability to produce plants with the knock-out of undesirable characteristics.

##### 2.1.3.1 Mechanism CRISPR/Cas system

The three stages of the CRISPR general mechanism are adaptation, expression, and interference. In the process of adapting, the bacteria take on the brief and distinctive protospacer sequences of the invaders and integrate them across nearby CRISPR loci in their genome. These sequences are recognized as novel CRISPR spacer sequences, which are added to the CRISPR array to create a memory of the invading organism. The next step is an expression in which the CRISPR locus is translated into pre-crRNA before being further processed into mature crRNAs, once the protospacer from the invasive organism is integrated into the CRISPR loci. In the last step, crRNA forms a complex with Cas protein, and the crRNA-Cas complex makes a complementary base pairing with the protospacer of the invader invading for the second time. Finally, crRNA-directed cleavage of the DNA follows with the aid of the Cas endonuclease protein. The system relies on two key molecules, Cas9 endonuclease, and a guide RNA, targeting a particular gene. gRNA (Spacer sequence) entails two RNA molecules, i.e., crRNA and tracrRNA, the former having the sequence complementary to the target DNA molecule and the latter acting as a binding partner for the Caspase 9 endonuclease enzyme. During exposure to invading virus or plasmid, short fragments of foreign DNA get integrated into CRISPR repeat-spacer array within host chromosomes as spacer DNA. They act as a marker of invading organisms. The transcription of this spacer DNA and enzymatic cleavage produces short CRISPR RNA (crRNA). During subsequent infection caused by the virus, hybridization occurs between crRNA and complementary foreign target sequence, marking it for sequence-specific cleavage by Cas9 endonuclease. The target site is recognized by a specific short conserved sequence motif, the PAM (Protospacer-adjacent motif) sequence, flanking between the target site of foreign invaders (Figure 1).

Makarova and her groups have immensely put their efforts into providing the evolutionary classification of the CRISPR-Cas system. In 2015 (Makarova and Koonin, 2015), the group emphasized 2 classes, 5 types, and 16 subtypes of the CRISPR system until recently, in 2020, where they provided an updated evolutionary classification including 2 classes, 6 subtypes, and 33 subtypes, based on the various effector modules and Cas protein compositions. The Class I CRISPR-Cas system comprises modules of several Cas proteins that bind and process the target as part of a complex called the cr-RNA-binding complex. A single, multidomain cr-RNA binding protein in the class II CRISPR-Cas system functions similarly to the entire class I system’s complex (Kato et al., 2022) Types 1, III, and IV are included in Class I, whereas Types II, V, and VI are included in Class II (Makarova et al., 2020).

The efficient delivery of the CRISPR/Cas9 complex to the target cell is an essential point to consider. It can be delivered in various forms, such as plasmid DNA, messenger RNA, or Ribonucleoproteins. Ribonucleoprotein (RNP), another emerging approach for genome editing, comprises of a Cas9 protein and a gRNA. Some of the critical points of RNP-based genome editing are its DNA/transgene-free genome editing approach which also ensures minimal off-target effects and being DNA-free, it offers reduced toxicity (Zhang et al., 2021).

##### 2.1.3.2 Genome editing using CRISPR/Cas9 technology in plants

The CRISPR-Cas9 system offers significant opportunities for genomic and epigenetic regulation in addition to its editing capabilities. The components of the CRISPR/Cas9 system Cas9 and sgRNA complex can serve as a scaffold to drive various effectors or markers to particular DNA sites. This feature of CRISPR-Cas9 has been implemented to modify the transcriptional level of gene expression, either to activate genes (CRISPRa) or to repress genes (CRISPRi) (La Russa and Qi, 2015). Even though being an important approach for genome editing, Double-Stranded Breaks open the door to off-target gene editing, hence unintended mutations. Therefore, other approaches, not including DSBs have been developed to escape these demerits. One such approach is using the dead Cas9 variant (dCas9) wherein the two catalytic domains are inactivated by point mutation and are inactive. They do not cause double-stranded breaks in the genome but still have strong binding efficiency to the target site in the genome. For this nature of dCas9, they are fused with other specific enzymes that can modify or bring the required changes in the genome. The two systems, CRISPRa/CRISPRi, also utilize nuclease-deactivated Cas9, whose catalytic domains are made disabled, and then fused to transcriptional modulators (Jensen et al., 2021).

##### 2.1.3.3 Base editing

Base editing, as the name suggests, is the tool whereby nucleotide base is either edited or modified with the guidance of sgRNA and the help of dCas9 protein fused with Activation-Induced Deaminases (AID) such as Cytidine deaminase or Adenine deaminase–based editors. Base editing is more effective because it does not call for double-strand breaks. Targeted editing for plants has utilized base editing.

Base editors- CBEs cause cytosine deamination to uracil, which then converts to thymidine by DNA replication or repair mechanism. CBEs have been immensely utilized for plant genome editing. As reported by Chen et al., CRISPR/CAS9-mediated base editing was performed for gain-of-functions mutation in Arabidopsis (Chen et al., 2017). Using Cytidine deaminase fused with Cas9 protein, Shimatani, and the group (Shimatani et al., 2017) were able to perform targeted base editing in rice and tomato for crop improvement.

Similarly, Adenine Deaminase (ABEs) fused with dCas9 causes base mutation from adenine to inosine, which can be further base paired with Cytosine. This cytosine pairs up with guanine in the newly synthesized strand, causing alteration in the dictating DNA. ABEs have already been used for genome editing, as reported in Arabidopsis, Rice, Wheat, etc.

##### 2.1.3.4 Epigenetic modifications

Gene expression can be changed by epigenetic alterations like DNA acetylation, methylation, or Histone modification. These epigenetic modifiers fused with the dCAS9 protein have been established as a better tool for generating epigenetic alterations (Pan et al., 2021). These modifications can alter the expression level of genes without changing the parental DNA sequence. Author Kang and his group (Kang et al., 2019) demonstrated the use of CRISPR/Cas9 to generate methylation at specific CpG sites and successfully targeted the Oct4 gene. Gallego-Bartolome with his group (Gallego-Bartolomé et al., 2018), altered the CRISPR/dCas9 SunTag system to target DNA demethylation in plants. In plants, the loss of 5 mC (5-methyl Cytosine) at the *FLOWERING WAGENINGEN* (FWA) promoter stimulates FWA expression, which is accountable for late-flowering. They achieved this by introducing the human TET1 (TEN-ELEVENTRANSLOCATION1) catalytic domain fused with CRISPR/dCas9.

##### 2.1.3.5 Prime-editing

Prime editing has been developed as a new tool kit for CRISPR-mediated genome editing by Anzalone et al. (2019). Like base editing, prime-editing does not rely either on DSBs or donor templates. Prime-editing involves three main components: Cas9 nickase, which is fused with Reverse Transcriptase enzyme, and a prime-editing guide RNA (pegRNA). pegRNA differs from other sgRNA in terms of its features: a sequence complementary to the target location of the template DNA at the 5′ end, Primer Binding Site (PBS) at the 3′ end, and the sequence carrying desired changes next to PBS. The Cas9 is drafted to the target location of DNA by the complementary guide sequence (5′ end) of the pegRNA and creates nicks at PAM-containing DNA strand. The RT template acts as a template for the synthesis of altered genetic information on the exposed 3′-OH group of target DNA as a result of the development of a nick, which causes the 3′ nicked end of the template strand to hybridize with the PBS of pegRNA. Hybridization of target DNA and RT produces either a 3′ flap having desired sequence or a 5′ flap having the original sequence (Wada et al., 2020). Endonucleases preferentially cleave the 5′ flaps, forming a DNA duplex with one edited strand with the desired alterations and another strand that is the original strand. This mismatch is either stably integrated by cellular replication or is corrected by a mismatch repair mechanism, both opening a gate for stable integration of the desired sequence into the genome (Kantor et al., 2020).

## 3 Genome editing for improving post-harvest quality in fruits, vegetables, and ornamental plants

### 3.1 Genome- editing in fruits

Agricultural modifications have shifted the paradigm of consumer acceptance. Traditional extrinsic visual-quality attribute awareness has moved to intrinsic attributes. Nutritional, functional, and physio-chemical factors of fruits and vegetables have been magnified in public awareness. These factors comprise the essential vitamins, minerals, phytochemicals, and antioxidant content traits introgressed. The dynamic genotypic and environmental factors determine the above attribute along with handling post-harvest. Post-harvest loss has been a major bottleneck, and a struggle to diminish the yield gap by improving the nutritional toolbox for genome editing to feed the growing population. The economic and social burden of the loss prompts the potential of the new-editing tools. It must be exploited alongside the conventional ones that have been limiting success. Due to their effectiveness and specificity of cleavage recognition sites, either by RNA-directed SDNs or protein-directed SDNs, site-directed nucleases (SDNs) utilized in plant editing technologies are frequently used to modify genomes. It includes meganucleases, ZFNs, TALENs, and CRISPR/Cas used to achieve random, predicted, or precise insertion. These all accomplish precise genetic alterations by causing deliberate DNA double-strand breaks (DSBs) (Tripathi et al., 2021). Additionally, CRISPR can avoid other obstacles, including sterility, self-incompatibility, high heterozygosity, a low frequency of recovering desirable alleles and features, and long life cycles that make conventional breeding operations more difficult or impossible to complete (Shipman et al., 2021). With the easy elimination of undesirable traits, the in-depth investigation of non-interference about the constitutive genome editing on other cellular functionality has increased. To avoid any pleiotropic changes on regulatory genes, Somatic mutations can be generated by special promoters in specific cells, tissues, and organs of the plant genome using the CRISPR- TSKO (tissue-specific knockouts), or TSGE (tissue specific gene editing) were preferred. It generated more accurate KO (knock-out) variants in plants. DJ3S, p54/1.0—Cassava promoters targeted into root of Carrot and Arabidopsis (Singha et al., 2022). Similar to this, another gene-editing method regulates the production of the Cas protein in fruits using an inducible chimeric transcription factor (XVE). The activator is an assembly of bacterial repressor LexA(X), activating domain VP16(V), and regulatory region of human estrogen receptor (E) (Brand et al., 2006). The estradiol-induced XVE is an inducible expression system used in transgenic apples, tobacco, *Arabidopsis* (Zuo et al., 2001)*,* and soybeans for reporter gene expression and overcoming certain limitations in other expression systems (Corrado and Karali, 2009). The outcomes of repair mechanisms chosen for genome editing applications that include site-directed nuclease-1 (SDN1) in kiwi-fruit (*ACC oxidase*) produce non-homologous end products after the cleavage of host DNA by CRISPR/Cas9. Non-homologous end joining (Brand et al., 2006). The outcomes of repair mechanisms for genome editing include site-directed nuclease-1 (SDN1), producing non-homologous end products after the cleavage of host DNA by CRISPR/Cas9. NHEJ causes indel mutations by gene silencing, gene inactivation, and gene knockout. SDN2 utilizes the plant Homology-Directed Repair (HDR) pathway to modify the gene’s activity by altering the sequence. The SDN2 techinique uses template DNA to produce an intended sequence modification at the DSB site. SDN3 technique uses a DNA insertion or substitution at a specific site in the DNA. Base and prime editing techniques are a type of SDN1 type. They can generate change in nucleotide without deploying a DSB or template DNA insertion in the locus targeted (Lin et al., 2020). The modifications of base and prime editing are comparable to the SDN2 technology. Oligo-directed mutagenesis (ODM) alters the genomic locus by targeted mutations. Successful application of ODM has been done on maize (Zhu et al., 2000), rice (Okuzaki and Toriyama, 2004), and oilseed rape (Gocal et al., 2015). The approach of SDN1, SDN2, and ODM targets mutation without the inclusion of exogenous DNA. But the SDN3 aims to target exogenous DNA insertion of various lengths (Modrzejewski et al., 2019). The delivery system of the SDNs includes PEG-fusion, electroporation, and biolistics, whereas, without the aid of any expression system. The chemically synthesized oligonucleotide used in ODM is supplied directly to the plant cell (Sandhya et al., 2020). However, the most reliable transformation for gene-editing is the Agrobacterium-mediated delivery in plants. Another approach to altering gene expression through DNA methylation has also been approached in orange and bell pepper (Cheng et al., 2018). Alongside genome editing, the combination of big data modeling and artificial intelligence (AI) monitoring has changed the course of field challenges. Most of the gene-editing tools has been researched on tomato and then progressed to achieve in several other fleshy fruits discovering numerous candidate gene targets (Rehman et al., 2022). Browning reactions in fruits post-harvest has also been triggering loss to the food industry. Potential research on natural bioactive compounds with anti-browning extracts (mangrove, green tea, thyme, pineapple) has been achieved to replace chemical additives by genome editing. An industrial approach for regulating the challenge has been worked through the activity control of the Polyphenol Oxidase (PPO) enzyme (Moon et al., 2020). Innovations targeting manipulation in the genome of plants have been extensively investigated and achieved at a spellbinding pace. ONM (Oligonucleotide directed mutagenesis), and ENs (Engineered nucleus) have been traditionally practiced and have been revolutionized with ZFNs, TALENs, EMNs, mi-RNA, and CRISPR. Modulation in the PPO and POD genes with miRNA, and CRISPR knock-out mutation reduced the browning activity in eggplant berries by 52% on the *SmelPPO4-5-6* gene (Maioli et al., 2020). Verification has been done by HRFA (High-Resolution fragment analysis) on the T1 phenotypic lines generated (Hamdan et al., 2022). The post-harvest storage also critically implicates the quality and shelf-life of freshly harvested fruits. The pathogenic microbes and pests in favorable growth conditions cause two types of deterioration: 1) physiological-softening, ripening, and senescence and 2) microbial deterioration by fungal or bacterial growth. Therefore, optimized storage conditions with CA (controlled atmosphere), DCA (dynamic controlled atmosphere), or ULO (Ultra-low oxygen) have been evaluated as chemical-free storage. Apart from the storage conditions, chemical treatments of pesticides have been used against the microbial activity. The implications of the hazardous by-products and residues have reduced their demand for usage. Comprehensive research has developed alternatives to chemical treatments using the physical-natural blend. Led light treatment, essential oil application, and edible coating (EC) are a few examples (Sánchez-González et al., 2011). EC is a semipermeable film over the fresh stock of perishable produce that has been catching attention for extending post-harvest life (Fawole et al., 2020). EC preserved fruit quality of ripening, softening, colour transformation, formation of sugar, and loss of organic acid by lowering the exchange of gaseous metabolic process in plants (Ziv and Fallik, 2021). Exploration of enzymatic inhibitors, nano and microencapsulation of the bioactive compounds is the need of the hour for implementing techniques to improve and utilize the natural extracts. Extensive broad-spectrum research on protein and gene expression profiles would also determine its biochemical, physiological, and receptor signaling activity pathways. This will help generate the required information about the natural extract’s target and through efficient statistical approaches, it can be effectively used on a large scale. Genome editing on fruits has dissected an imminent network of signaling and biological pathways that is anticipated to aid in investigating hitherto undiscovered genes inducing positive post-harvest phenotypes (Table 1).

**TABLE 1:** List of the targeted genes that are modified by the CRISPR/Cas9 tool in fruits, vegetables, and ornamental plants.

Crop	Gene	Method	Gene function	Character	References
Vegetables					
Sweet potato	GBSSI	CRISPR/Cas9	Granule-bound starch biosynthesis	decreased amylose content	Wang et al. (2019)
	SBEII	CRISPR/Cas9	Starch branching for amylopectin	decreased amylopectin content; increased amylose	Wang et al. (2019)
Potato	PP02	CRISPR/Cas9	Converts phenolic substrates to quinones	decreased browning	González et al. (2020)
	16DOX	CRISPR/Cas9	Steroidal glycoalkaloid biosynthesis	decreased steroidal glycoalkaloid content	Nakayasu et al. (2018)
	SSR2	TALEN	Sterol side chain reductase 2	Reduced steroidal glycoalkaloids	Yasumoto et al. (2019)
	PPO5	TALEN	Polyphenol oxidase	Reduced black spot, enzymatic darkening and discoloration in potato tubers	Moon et al. (2020)
	Vinv	TALEN	Accumulation of reducing sugars which cause acrylamide accumulation	reduced levels of acrylamide	Clasen et al. (2016)
	Vinv	TALEN	Hydrolyzes the sucrose produced from starch breakdown into one molecule of glucose and one of fructose	Improved cold storage and pricessing time	Clasen et al. (2016)
	StPPO2	CRISPR-Cas9	Catalyzes the oxidation of phenolic compounds into compounds into quinones (highly reactive form)	Reduction in enzymatic browning	González et al. (2021)
*Solanum tuberosum*	Polyphenol Oxidases (PPOs)	CRISPR/Cas9 system	Causes oxidative browning	Lower the enzymatic browning in tubers	González et al. (2020)
*Solanum lycopersicum*	ALC (Alcobaca)gene	CRISPR/Cas9 system	Involved in ripening process	Improve shelf life	Yu et al. (2017)
	LeMADS-RIN gene	CRISPR/Cas9 system	Regulates ripening	Lower ripening and ethylene production	Jung et al. (2018)
	VInv (vacuolar invertase gene)	TALEN	Reducing sugar accumulation in cold-storage	Improve cold storage and processing by minimizing the production of reduced sugar	Clasen et al. (2016)
	Expansin 1	Site-directed mutagens	Significantly enhance root-network and root-biomass, promote cell wall loosening	Fruit ripening	Brummell et al. (1999)
Tomato	GABA-TP1, GABATP2, GABA-TP3, CAT9 and SSADH	CRISPR/Cas9	Essential genes for the γ-aminobutyric acid (GABA) pathway	reduced concentration of γ-aminobutyric acid	Li et al. (2018)
	MYB12	CRISPR/Cas9	Flavonoids Metabolic Pathways	Pink tomatoes	Deng et al. (2018)
	CRTISO or PSY1	CRISPR/Cas9	Carotenoids Metabolic Pathways	orange tomatoes and yellow tomatoes, respectively	Dahan-Meir et al. (2018)
	SlANT1	TALEN and CRISPR	Anthocyanin biosynthesis	purple tomatoes	Čermák et al. (2015)
	SGR1, LCY-E, Blc, LCY-B1, and LCY-B2	CRISPR/Cas9	Carotenoids Metabolic Pathways	5.1-fold increase in the lycopene content	Li et al. (2018c)
	SlDDB1, SlDET1, SlCYC-B	Target-AID	Carotenoids Metabolic Pathways	increased carotenoid, lycopene, and β-carotene	Hunziker et al. (2022)
	SlGAD2 and SlGAD3	CRISPR/Cas9	Aminobutiric acid Metabolic Pathways	sevenfold to 15-fold increase in GABA accumulation	Nonaka et al. (2017)
Tomato	SlANT2, SlAN2-like	CRISPR/Cas9	Anthocyanin biosynthesis	Decreased anthocyanin content	Yan et al. (2020)
	HYS	CRISPR/Cas9	Anthocyanin biosynthesis in response to light	Decreased anthocyanin content	Qiu et al. (2019)
	FLORAL4	CRISPR/Cas9	Regulates phenylalanine-derived volatiles in fruit	Increased phenylalanine-derived volatile content	Tikunov et al. (2020)
	RIN	CRISPR/Cas9	Ripening control *via* ethylene	decreased volatile organic compounds	Tikunov et al. (2020)
	L1L4	ZFN	Metabolite pathway	SSC, fiber, fructose, ascorbic acid, total	Gago et al. (2017)
	cycB	CRISPR/Cas9	Metabolite pathway	phenol, carotene, oxalic acid, high lycopene content	Zsögön et al. (2018)
	RIN	CRISPR-Cas9	Inhibit ethylene synthesis and specific biochemical processes related to fruit ripening Inhibit ethylene synthesis	Lower ehylene content in mutant lines and delayed fruit ripening	Jung et al. (2018)
	ALC	CRISPR-Cas9	Inhibit ethylene synthesis (SN1 is an insertion of an actual inhibitor gene ALC)	Mutants with longer self-life	Lü et al. (2018)
	ALC	CRISPR-Cas9	Inhibit ethylene synthesis	Mutants with longer self-life	Yu et al. (2017)
	SBP-CNR &NAC-NOR	CRISPR/Cas9	Transcription factor of ripening genes	Mutants with delayed non-ripening phenotype	Gao et al. (2019)
	Ripening inhibitor gene, SIMADS-RIN	CRISPR-Cas9	Ethylene production hence fruit ripening	regulating ethylene synthesis, ripening, and regulate shelf-life	Ito et al. (2015)
Wild tomato	GGPI	CRISPR/Cas9	Vitamin C metabolism	increased vitamin C content	Li Y. et al. (2018)
Eggplant	PP04, PPOS, and PP06	CRISPR/Cas9	Converts phenolic substrates to quinones	decreased browning	Maioli et al. (2020)
Carrot	F3H	CRISPR/Cas9	Anthocyanin biosynthesis	Decreased anthocyanin content	Klimek-Chodacka et al. (2018)
Chinese kale	BoaCRTISO	CRISPR/Cas9	Carotenoid biosynthesis	yellow color of Chinese kale with improved market prospects	Sun et al. (2020)
Lettuce	LsGGP2	CRISPR/Cas9	Deleted uORFs of LsGGP2 to increase the translation of mRNAs	increased oxidation stress tolerance and ascorbate content	Zhang H. et al. (2018)
Pomegranate	PgUGT84A23 and PgUGT84A24	CRISPR/Cas9	UDP-dependent glycosyltransferases (UGTs) enzymes with overlapping activities in ß-glucogallin biosynthesis	unique accumulation of gallic acid 3-0- and 4-0-glucosides	Chang et al. (2019)
*Brassica oleracea var. capitata*	BoPDS gene (Phytoene desaturase gene)	CRISPR/Cas9 system	Male sterility associated gene	Albino-phenotype (Improve variety)	Ma et al. (2019)
*Capsicum annuum L.*	C. annuum ethylene-responsive factor 28 (CaERF28)	CRISPR/Cas9 system	Annuum anthracnose pathogen *Colletotrichum truncatum* resistance	Anthracnose resistance	Mishra et al. (2021)
Fruits					
Banana	MaGA20ox2	CRISPR/Cas9 system	Regulation of gibberellin production	MaGA20ox2 Semi-dwarfing	Shao et al. (2020)
	PDS	CRISPR/Cas9 system	Carotenoid biosynthesis	Albino	Naim et al. (2018)
	PDS	CRISPR/Cas9 system	Carotenoid biosynthesis	Albino	Kaur et al. (2018)
Apple	TFL1 & PDS	CRISPR/Cas9 system	Regulate flowering time and meristem development	Early flowering, and albino phenotype	Charrier et al. (2019)
	DIPM-1, 2, and 3	CRISPR/Cas9 system	Fire blight susceptibility	Resistance for Fire blight disease	Malnoy et al. (2016b)
	PDS	CRISPR/Cas9 system	Carotenoid biosynthesis	Albino phenotypes	Nishitani et al. (2016)
Strawberry	PDS	CRISPR/Cas9 system	Carotenoid biosynthesis pathway	Albino phenotypes	Wilson et al. (2019)
	AP3 (APETALA3)	CRISPR/Cas9 system	Floral organ development	Flowering control	Martín-Pizarro et al. (2019)
	FvARF8 (Auxin response factor 8) and FveTAA1 (Auxin biosynthesis gene)	CRISPR/Cas9 system	Control sensitivity to the plant hormone auxin	FvARF8 (Auxin response factor 8) and FveTAA1 (Auxin biosynthesis gene) Auxin biosynthesis	Zhou et al. (2018)
Grape	VvWRKY52	CRISPR/Cas9 system	Carotenoid biosynthesis pathway	Resistance for Botrytis cinerea	Wang X. et al. (2018)
	PDS	CRISPR/Cas9 system	Floral organ development	Albino phenotypes	Nakajima et al. (2017)
	PDS	CRISPR/Cas9 system	Control sensitivity to the plant hormone auxin	Albino phenotypes	Ren et al. (2019)
	IdnDH	CRISPR/Cas9	Tartaric acid biosynthesis	decreased tartaric acid content	Ren et al. (2016)
*Musa acuminate*	aminocyclopropane-1-carboxylase oxidase (MaACO1), MaMADS1 and MaMADS2	CRISPR/Cas9 system	Control ethylene production	Delayed ripening, increased shelf life	Elitzur et al. (2016)
Vitis vinifera L.	L-idonate dehydrogenase gene (IdnDH)	CRISPR/Cas9 system	Control biosynthesis of tartaric acid	Targeting tartaric acid pathway	Ren et al. (2016)
Ornamental					
*Petunia*	1-aminocyclopropane-1-carboxylate oxidase1	CRISPR/Cas9 system	Ethylene biosynthesis	Flower longevity enhance	Xu et al. (2020)
	PhHD-Zip	Virus-induced gene silencing	Regulate flower senescence	Flower senescence	Chang et al. (2014)
*Petunia inflata*	PiSSK1	CRISPR/Cas9 system	Recogniton of non-self S-RNase during cross-compatible pollination	Pollination	Sun and Kao (2018)
*Torenia fournieri*	CYCLOIDEA–RADIALIS (TfCYC1, TfCYC2, and TfRAD1)	CRISPR/Cas9 system	Transcription activator regulate flower morphology	Regulates petal shape and pigmentation	Su et al. (2017)
	flavanone 3-hydroxylase (F3H) gene	CRISPR/Cas9 system	Flavonoid biosynthesis	Flower color	Nishihara et al. (2018)
*Phalaenopsis equestris*	MADS genes	CRISPR/Cas9 system	Flavanoid pathway	Flower initiation and development	Tong et al. (2020)
*Phalaenopsis orchid*	CymMV coat protein	RNA-mediated gene silencing	Ethylene biosynthesis	Cymbidium Mosaic Virus resistance	Liao et al. (2004)
*Lilium*	LpPDS gene	CRISPR/Cas9 system	Regulate flower senescence	Phenotype	Yan et al. (2019)
*Cyclamen persicum Mill. (cyclamen*)	flavonoid 3′, 5′-hydroxylase	Antisenec suppression	Recogniton of non-self S-RNase during cross-compatible pollination	Flower color	Boase et al. (2010)
Petunia	PhACO	CRISPR-Cas9	Catalyzes aminocyclopropane-1-carboxylic acid to ethylene in ethylene biosynthesis pathway	Reduction in ethylene content and longer flower longivity time	Xu et al. (2020)

### 3.2 Genome-editing in vegetables

There is currently a great deal of attention on the health advantages of consuming vegetables of the expansive array of nutrients found in them, such as vitamin supplements, minerals, antioxidants, dietary fiber, and phytochemical compounds (Septembre-Malaterre et al., 2018). Vegetables, whether consumed fresh or in processed form, defend against a wide range of non-transmitted diseases and assist in the reduction of such food insecurity in underdeveloped countries (Desjardins, 2014). Food losses and waste reduction are crucial to a country’s food security as intensive and extensive farming. Losses reduce the availability of food, contributing to food insecurity and waste food generating unnecessary emissions of CO_2_ as well as a failure of the food’s economic value. As a result, limiting post-harvest losses in agricultural products is critical for food availability and security in developing countries. Post-harvest losses are losses in the nutritional value, seed viability, and selling price of food that occur along the entire food supply chain, from harvest to consumption (Ghosh et al., 2011). Genome editing allows for the acquisition of multiple homozygous mutations in a single generation without foreign DNA, opening up new avenues for genomics and the breeding of crops and quality traits in vegetables. The tomato and potato have attracted the most focus on genome editing research and implementation.

The ability to alter important characteristics in cucumber, watermelon, lettuce, and broccoli has been demonstrated (Cardi et al., 2017). In tomato seeds, gene LEAFY-COTYLEDON1-LYKE4, that encodes the β -subunit of the transcription factor Y, is damaged by the transiently expressed zinc finger nucleases (ZFNs). Plants with mutations displayed a wide range of morphological changes as well as the expression of genes involved in hormone metabolism, especially the ethylene biosynthetic pathway. No off-target changes were found in the closely linked *L1L2* and *L1L3* gene sequences (Hilioti et al., 2016). GABA (Y-aminobutyric acid) is a chemical that promotes health and has received a lot of attention in traditional tomato breeding research. Using genome editing, multiple GABA pathway genes were modified, resulting in a 19-fold increase in GABA content in *Solanum lycopersicum*. Resistance to diseases through genetic modification is another new area of study in vegetables rapidly expanding as the CRISPR/Cas system as a genome editing tool. Genetically modified by deletion of 48 base pair in homozygous layout in MLO1 locus, tomato plants generated which showed resistance to powdery mildew disease affected by *Oidium neolycopersici* (Erpen-Dalla Corte et al., 2019)*.* Editing the *SIDMR6-1* gene also produced bacterial disease resistance (downy mildew resistance 6 gene) (Tripathi et al., 2020). The shift in the recent paradigm of abiotic stress tolerance research includes more horticulture crops for genetic modification. Significant advances have been made in the improvement of biotic stress tolerance (fungal infection and virus resistance) as in tomatoes and cucumbers (Jain, 2015; Shi et al., 2017). Multiple research studies have pivoted the potential functions in plant protection against abiotic and biotic stresses, as well as improving fruit quality, plant architectural features, and storage stability (Kulus, 2018). The N′ and C′ ends of eIF4E-were targeted by CRISPR/Cas in cucumbers by agrobacterium-mediated transformation, leading to resistance to cucumber vein yellow virus, pumpkin mosaic virus, and papaya ring spot mosaic virus (PRSV-W) (Chandrasekaran et al., 2016). Numerous diseases are brought on by fungi, which can significantly reduce crop output and quality. For example, downy and powdery mildew significantly reduce tomato production (Borrelli et al., 2018). CRISPR/Cas9 knockout of DMR6 homologous genes in tomatoes demonstrated resistance to *Pseudomonas syringae*, Phytophthora, and Xanthomonas spp (de Toledo Thomazella et al., 2016). Climate change is making crops more susceptible to abiotic stress. The invention of CRISPR/Cas9 has accelerated the production of new varieties. Several developmental pathways that are mediated by brassinosteroids (BR) involve the brassinazole-resistant 1 gene (BZR1). The agrobacterium-mediated CRISPR-mediated mutation in BZR1 (engaged in a variety of brassinosteroid (BR)-mediated developmental processes) inhibited the induction of RESPIRATORY BURST OXIDASE HOMOLOG1 (RBOH1) and the production of H_2_O_2_. Exogenous H_2_O_2_ restored heat tolerance in tomato bzr1 mutants (Yin et al., 2018). To develop larger fruit size than the WT fruits, Cold Spring Harbor Laboratory edited the tomato CLAVATA-WUSCHEL (CLV-WUS) stem cell gene CLV3’s promoter region using the CRISPR/Cas9 technique (Li T. et al., 2018). High concentrations of steroidal glycoalkaloids (SGAs) give potato tubers a foul flavor and make them dangerous to consume. St16DOX (steroid 16-hydroxylase) was removed from the potato SGA biosynthetic pathway using CRISPR/Cas9, producing SGA-free potato lines (Nakayasu et al., 2018).

### 3.3 Genome-editing in ornamental plants

Ornamental plant production is linked with various features and markets like cut flowers and decorative vegetation, interior and exterior houseplants, window ledges, bulbous and nursery plants. In the scenario of ever-increasing demand, the floriculture industries require advancements and more different kinds with privileged qualities. The major objectives of ornamental breeding programs include establishing uniqueness in a blooming plant, color diversity, and perfumes, as well as inflorescence development by enhancing the flower count, altering the duration and longevity of flowering. Flower colors are the most impactful for their economic interest of any characteristic. Yellow cyclamens, red iris, blue chrysanthemums and roses, for example, are not found in nature due to the absence of pigment pathways (Tanaka et al., 2009). New decorative plant cultivars have been produced using a variety of breeding techniques. Apart from enhancing plant architecture and disease resistance, traditional plant breeding practices like crossbreeding and mutation breeding have been employed to produce various patterns and colours in plants. The majority of present methods for ornamental plants are extremely heterozygous, which produces a multifaceted transmission of genetic traits and polyploidy with several limits and drawbacks (Azadi et al., 2016; Sharma and Messar, 2017). Only a few countries allowed genetically modified (GM) ornamental plants commercially. For instance, the blue rose “ApplauseTM” and the violet carnation “MoondustTM,” both of which are genetically modified (GM) to hoard delphinidin-based anthocyanins, were developed and commercialized in the global flower market by the Japanese company SUNTORY and the Australian biotechnology company Florigene. However, consumers prefer genetically modified crops to genetically engineered ornamental plants (Nishihara and Nakatsuka, 2011). The first genome of an ornamental plant to undergo CRISPR/Cas9 modification was *Petunia hybrida* (Subburaj et al., 2016). The NITRATE REDUCTASE (PhNR) gene exhibited maximal targeted mutagenesis by direct administration of designed RNA-guided endonuclease (RGEN) ribonucleoproteins (RNPs) induced in Petunia protoplast cells. This has been made possible by the quick advancement of genome editing tools like ZFN, TALENs, and CRISPR/Cas9. These methods also enable a comprehensive understanding of the metabolic activity of annotated genes in newly sequenced and assembled genomes. Flower colour modification is now widely used in many plant species through genome-editing tools. Mutagenesis in the dihydroflavonol-4-reductase (DFR) gene of *Ipomoea nilotica* using CRISPR/Cas9 resulted in the first floral colour alteration in higher plants (Watanabe et al., 2017). Watanabe modified the carotenoid cleavage dioxygenase 4 (CCD4) gene using CRISPR/Cas9 to produce mutant Ipomoea plant. CRISPR/Cas9 approaches are used to target multiple MADS genes involved in floral organ development in the Phalaenopsis orchid (Tong et al., 2020). The chrysanthemum is one of the most well-known and important floricultural crops. Despite a complete genome sequence, due to the enormous genomic size and ploidy stages with a higher fraction of repetitive sequences, CRISPR/Cas systems will probably be challenging to implement. The CRISPR/Cas9 system functions in this plant, according to just one study (Kishi-Kaboshi et al., 2017) (Table.1).

## 4 Genetic engineering for the nutritional enhancement and shelf-life

### 4.1 Nutritional enhancement

The gradual increase in customer demand for extrinsic quality characteristics in fruits and vegetables, such as size, color, texture, aroma, storage life, etc., has been considered crucial. With increased public awareness, nutrition enhancement became economically significant. The commercialization of genetically engineered approved horticulture crops included virus-resistant papaya, squash variety, non-browning apple, fleshy pineapple, insect tolerant eggplant (BT brinjal), *Brassica oleracea*-(*BolC.GA4. a* gene), Tomato-(*PSY1,Mlo,GABA-TP1,TP2, SIAGL6* gene), Potato- (*StMB44, StALS1* gene) and *Lactuca sativa-(BIN2* gene). The demand for parthenocarpic fruits and their socio-economic impacts accelerated the potential for developing genome editing. High-quality cultivars have been developed by silencing genes involved in signaling, knockout technique, reverse genetics, transgenesis, etc. The notable accomplishments through CRISPR/Cas9 techniques have brought about successful trait modification through mutation for enhancing nutritional gains. Mutations included loss of function in tomato (*SIALC, SIFUL1, Pectate lyase*), banana (*MaLCY epsilon, MaPDS*), apple (*MdPDS, MdDIMP4*), grapes, kiwi fruit (*AcPDS, AcCENs*), soybean (*GmPRR3b, 37, GmFT2a*), potato (*StPPO*), strawberry (*FaTM6*), gene or promoter insertion, cis-regulatory alleles in tomato (*SICLV3, lycopene beta cyclase*)*,* citrus (*CsLOBs*) frame-shift mutation, gene replacement in tomato (*SIALC*), etc. The modifications in the aforesaid horticultural crops included increased flower and fruit size, fruit ripening, inflorescence branching, enhanced ascorbic acid synthesis, fortification of beta-carotene (banana), the transformation of perennial to annual (kiwi fruit), decreased tuber-browning and enhanced quality of berry (strawberry) (Tiwari et al., 2021). In an elegant research study, it has been published that the flavor of fruit directly modulates certain organic acids that influence organoleptic characteristics. Hence improving or minimizing them has a direct impact on the general character of a fruit (Sweetman et al., 2009). Acidity, which is quite a significant trait of the fruit harvest, has a direct impact on fruit quality. But it is especially critical for later processing. Hence, early-ripening apples often have high levels of acidity and low levels of sugar, which lowers the market demand for fresh consumption and leads to a low proportion of total organic acids to total carbohydrates. Numerous recent research in the fields of omics and quantitative trait loci (QTLs) (Xu et al., 2012) revealed a significant cellular synchronization between nutrition, post-harvest fruit quality, and shelf-life variation (Lin et al., 2016). The effectiveness of UV-C usage to combat the post-harvest impacts on early-ripened fruits produced at room temperature include ripening delay, senescence, preserving the significant ratio of fruit firmness, biosynthesis of flavonoids, phenolic content, enhanced antioxidant, and defense responsive molecules (Artés et al., 2009). The different doses for the UV irradiations have a marked positive impact by lowering the acidity-to-sugar ratio of various fruits and vegetables to preserve them post-harvest. UV-C treatment has been well-established on apples, mango, strawberry, peach, tomato, etc., promoting the flavour. The irradiations generate malate degradation-improving the quality of fruit, plant defense response mechanism against microbes, activating the gene regulation for disease resistance, enhancing salubrious phytochemical content, and regulating the proportions of antioxidant ratio on exposure to the shorter wavelength of UV-C. For successful implementation of post-harvest storage conditions to enhance nutritional benefits, the various horticultural produce effect is being noted. It includes the specific plant parts, developmental stages targeted for treatment, doses used, cultivar specificity, and harvesting time of the fruits and vegetables.

### 4.2 Shelf life

Postharvest loss and waste are becoming increasingly unsustainable as global horticulture crop production is inadequate for meeting human nutritional needs. Postharvest loss is unintentional. It outlines the sporadic losses that farmers occasionally sustain at the hands of consumer, including physical harm, internal bleeding, premature spoilage, and bug damage, among other things. Ornamental popularity has exploded recently, with an average worth of $16 billion in 2015 (Kader, 2004; Porat et al., 2018). Ornamental crops have a high moisture content, and the cold-chain process results in a loss of up to 50% of farm value (Pranuthi et al., 2018). Value decreases by 15% for each additional day spent on transit. Furthermore, the vase-life of ornaments is usually just 10–12 days after consumer purchase (Mamias, 2018). So, it is important to move things along a cold chain quickly. Reduced respiration rate is achieved by using cold temperatures, which also increases shelf life. As vegetables and fruits travel from farmer to customer, various factors (processing, storage, and transportation conditions) contribute to such a deteriorative process. Understanding where food supply chain losses occur is critical for determining possible causes and improving best post-harvest practices (Gustavsson et al., 2011). Temperature, moisture content, level of ethylene hormone, and the storage proportion of O_2_ to CO_2_ must be managed to ensure yield shelf-life and quality (Kader, 2004). Infectious agent’s invasion attempts on harvested products are likely to caramelise the fruit ingredients, resulting in diseased or spore-covered fruits and their metabolic by-products. The products are unaesthetic and non-edible due to the acidic, bitter, foul-smelling, and toxicants produced (Encinas-Basurto et al., 2017).

Fruits that are overripe or underripe are more vulnerable to physiological disorders. Sometimes entire products are discarded as they are unsafe to eat (ömer AZABAĞAOĞLU, 2018). Incorrect cultural practises such as cold snaps, weather, water stress, heavy rainfalls, pathogens, physiological disorders, plant health, safeguards, water management, fertilization, and cutting possibly cause fruit and vegetable damage during the preharvest period. Harvesting period loss (4%–12%) is due to inaccurate harvesting time estimation, harvesting at the wrong time, inappropriate harvesting procedures implementation, and not applying pre-cooling to fruits such as cherries during the harvesting process (Özdemir et al., 2003). According to the International Refrigeration Institute (IIR), 23% of food waste in developing countries is generated when cooling systems are not used [(Dupont et al., 2009), 2009]. Constant cold storage ensures that the product reaches the consumer in pristine condition. The lack of adequate storage facilities is the leading cause of the degradation of both qualitative and quantitative factors of food production from food harvest to consumption level in underdeveloped countries (Tatlıdil et al., 2013). Fresh produce changes cannot be avoided, but they can be reduced with preventative measures, including cold temperature, relative humidity, appropriate transportation and packaging, and others (Ahmad and Siddiqui, 2015). The most popular ornamental plants, such as roses, liliums, lisianthus, chrysanthemums, and carnations, demonstrated varying levels of ethylene sensitivity and flower longevity. The use of breeding methods in conjunction with ethylene screening was only partially successful. It is possible to expand the life of ornamental plants by using molecular techniques (Netam, 2018). Gene-editing of 1-aminocyclopropane-1-carboxylate oxidase1 (PhACO1) gene which encodes for ethylene producing enzyme *via* CRISPR/Cas9 method resulted in decreased ethylene and delayed petal senescence in the petunia cultivar “Mirage Rose” (Xu et al., 2020). Mutations in the EPHEMERAL1 (EPH1) gene, which encodes the major regulator for petal senescence as a NAC transcription factor, resulted in delayed petal senescence in Japanese morning glory (Ipomoea nil “Violet”) plants (Shibuya et al., 2018). MaACO1 mutated through CRISPR/Cas9 system produced less ethylene and had a longer shelf life in the ripening stage (Hu et al., 2021). To date, the CRISPR/Cas9 gene-editing approach has already been effectively implemented in many fruit crop species, such as climacteric ripening species, like apples (Malnoy et al., 2016b; Nishitani et al., 2016), bananas (Kaur et al., 2018), kiwifruit (Wang Z. et al., 2018)), and non-climacteric ripening species, such as sweet orange (Jia and Wang, 2014), Duncan grapefruit (Jia et al., 2016), grapevine (Malnoy et al., 2016b), watermelon (Tian et al., 2017), cucumber (Chandrasekaran et al., 2016), and cultivated strawberries (Martín-Pizarro et al., 2019).

## 5 Biotic and abiotic stress resistance in horticultural crops using the CRISPR/Cas9 technology

### 5.1 Abiotic stress

Abiotic stress, which includes dehydration, soil salinity, and high heat, poses serious risks to crop development, yield, and quality (Sharma and Gayen, 2021). The effect of adverse conditions on the plant is more common in tropical regions than temperate regions. High temperature creates more threat in tropical countries. Drought stress is another abiotic stress limiting crop development and production, which is continuously increasing due to global warming. Crop yields are becoming more and more susceptible due to unfavorable climate changes, deteriorating soil health, and declining air quality. Researchers are attempting to create a transgenic crop that can produce more and easily tolerate severe and variable conditions. CRISPR/Cas9 technology has been implemented to increase crop yield. Abiotic stress, such as drought, soil salinity, and high temperature, create major problems for the growth and development of the crop, drastically lowering agricultural production and quality. Resistance to the abiotic stress created by the many genes is a complex trait. With the use of nucleases that cause double-strand breaks by providing a new method for editing genes in molecular biology, which transformed the field of genome editing. ZFNs are credited as the 1st genome editing technique that used programmable nucleases to make a significant advancement in the field of genome engineering (Chandrasegaran and Carroll, 2016). After a while, TALENs (Transcription activator-like effector nucleases), which are entirely dependent on TALEs of the bacteria, additionally broaden genome engineering potential. TALENs were quickly adapted to approximately 40 different types of species for genome editing (Chandrasegaran and Carroll, 2016). Scientists worldwide have turned their attention to the CRISPR/Cas9 technologies since their discovery because of their numerous advantages over TALENs and ZFN (Mao et al., 2013). Unlike the previous technology, TALENs and ZFN, which use a protein motif to identify the target, CRISPR/Cas9 is based on RNA-DNA recognition to generate the double-strand break. Apart from this, there are many benefits of the CRISPR/Cas9 technology over the TALENs, and ZFN 1) Ease of the target design 2) ability to directly inject RNAs encoding the Cas9 protein and gRNA (guide RNA) to cause mutation, and 3) create targeted mutations in numerous genes in a single event because to the simplicity of multiplexing (Ma et al., 2015; Malzahn et al., 2017). A new genetic engineering tool is precise genome editing for crop improvement. Multiple techniques such as TALENs (Christian et al., 2010; Li et al., 2011; Miller et al., 2011), ZFNs (Maeder et al., 2008; Sander et al., 2011), RGENs (RNA-guided nucleases), and CRISPR/Cas9 (Jinek et al., 2012; Cong et al., 2013; Mali et al., 2013), all these types of technology developed for the targeted genome editing. CRISPR/Cas9 technology-based genome editing in the crop can be used to modify almost any sequence to show its function in the organism’s genome. CRISPR/Cas9 generated *SIMAPK3* participates in tomato dehydration resistance by preventing oxidative damage to the cell wall generated by stress, further adjusting the transcription of genes linked to drought stress (Wang et al., 2017). Recently, CRISPR/Cas9-generated genome engineering for high-temperature resistance has been achieved by focusing on an S gene, *SIAGL6* (SIGAMOUS-LIKE 6), in *S. lycopersicum*, which has a significant role in the improvement in fruit quality against high-temperature stress (Klap et al., 2017). In tomatoes involvement of the auxin in salinity stress and osmotic stress resistance and the role of the ARF4 (auxin response factor 4) providing the resistance and confirm the use of the CRISPR/Cas9 tools to develop tolerant plants (Bouzroud et al., 2020). In tomato genome editing by the CRISPR/Cas9 tools under the abiotic stress in various genes such as *SIMAPK3* (Kong et al., 2012), *SILOX*, *SIGST*, *SIDREB* (Wang et al., 2017), *SILBD40* (Liu et al., 2020), *SINPR1* (Li et al., 2019), and *SIMAPK6* (Li et al., 2021), under the abiotic stress condition. CRISPR/Cas tools were used to generate mutation in lettuce plants to understand better the mechanism of NCED4 (Bertier et al., 2018). During heat stress, CRISPR/Cas9 technology was used for silencing *SIMAPK3* in tomatoes (Yu et al., 2019). This technique is used to increase the expression level of the *ARGOS8* gene (a negative regulator of the ethylene response) to develop drought resistance maize crops, and the *ARGOS8* gene promoter changed into *GOS2*. These traits had a major role in the enhancement of grain yields during drought stress conditions (Hirai et al., 2007). Overexpression of the biosynthesis of the melatonin gene in plants was identified as an abiotic stress resistance (Byeon and Back, 2016; Antoniou et al., 2017). Currently, the CRISPR/Cas9 genome engineering technique further expands the application of these tools to genome-wide screening for the improvement of the desired trait (Rodríguez-Leal et al., 2017; Mahas and Mahfouz, 2018). Gain- or loss-of-function mutants can result from precise base alterations made by the CRISPR/Cas9 system at desired gene locations. There is no doubt that CRISPR/Cas technology might replace conventional crop breeding methods. It depends on locating plant populations with genetic differences to produce desired features in crop cultivars.

The gRNA sequencing method can be used to identify novel allelic variations that match a specific desirable trait that could be introduced into plant populations by this base editing technique (Eid et al., 2018).

### 5.2 Biotic stress

Biotic stress, such as insects, viruses, fungi, and bacteria, can attack plants and cause severe damage (Langner et al., 2018). CRISPR/Cas9 tools have been developed to obtain disease-tolerant plants (Arora and Narula, 2017). CRISPR/Cas technology is a beneficial method for creating stable transgenic lines. Following the development of CRISPR/Cas technology against bacterial, viral, and fungal infections, which result in significant losses in plants. Despite the information of technological advances being already reported in CRISPR/Cas9 technology for the fruits, vegetables, and ornamental plant improvement, not much more progress has been made towards the uses of CRISPR/Cas9 genome engineering tool for biotic stress resistance in fruits, vegetables, and ornamental crops. The response of the plants toward biotic stress is more complicated; during the biotic stress circumstances, several crucial genes are overexpressed and down-expressed.

For the inactivation of the DMR6 ortholog in *Solanum Lycopersicum*, researchers used the CRISPR/Cas9 system. They discovered that mutations in the dmr6 gene exhibit disease tolerance against various pathogens, including species Xanthomonas, *Phytophthora capsica*, *and P. syringae*, without causing any discernible adverse effects (de Toledo Thomazella et al., 2016)*.* It has been demonstrated that tomato MAPK3 (mitogen-activated protein kinase-3) gains the potential to Botrytis cinerea (Zhang S. et al., 2018)*.* Scientists used CRISPR/Cas9 to develop *Solanum Lycopersicum* JAZ2 (Jasmonatezim domain protein 2) repressors lacking C-terminal jasmonate associated domain (JAZ2Δjas). These repressors provide tolerance against the *P. syringae*, indicating CRISPR/Cas9 technology for the development of resistance fruit and its implementation in agriculture (Ortigosa et al., 2019)*. In* Vitis vinifera*,* knockout of the WRKY5 by the CRISPR/Cas9 technology encoding a TF that has a significant role in biotic stress response, WRKY5 exhibits disease tolerance against *B. cinerea* (Wang X. et al., 2018)**.** The CRISPR/Cas9 technology is crucial for the fruits such as apples (*Malus domestica*) and grapes (Vitis vinifera), which have long life cycles and generation times. Numerous papers have described the use of CRISPR/Cas9 so far with fruits like *Citrus reticulate* (Jia and Wang, 2014), grapes (Malnoy et al., 2016a; Ren et al., 2016; Wang et al., 2016), apples (Malnoy et al., 2016a; Nishitani et al., 2016), *Solanum tuberosum* (potato) (Andersson et al., 2017), banana (*Musa balbisiana*) (Tripathi et al., 2019), cucumber (Chandrasekaran et al., 2016), papaya (Gumtow et al., 2018), citrus (Jia et al., 2016; Peng et al., 2017). The fruits, vegetables, and ornamental plant production and growth are exposed to continuously increased risks of biotic stress. The CRISPR/Cas9 technology is used in applications to obtain new germplasm resources rapidly. As fruits, vegetables, and ornamental plants are sensitive to biotic stress, it can make achieving optimal yield difficult, emphasizing the significance of creating stress-resistant crops.

## 6 The difficulty, challenges, and solutions

The application of CRISPR/Cas9-based genome editing technology in fruits, vegetables, and ornamental plants has specific significant methodological challenges that need to be addressed to appreciate the uniqueness of this technique fully. The following are some main problems with the CRISPR/Cas9 genome editing technique: 1) The first prerequisite for beginning genome editing work is the species’ whole genome information. Although notably in many crops, it is not available, which is more complicated and severely restricts the applicability of CRISPR/Cas9. 2) The reproducible and efficient gene transfer methodology containing particle bombardment, *in vitro* generation protocol, Agrobacterium-mediated transformation, due to their resistance to plant tissue culture, many fruits, vegetables, and ornamental plants have not been investigated for PEG-mediated transformation, etc. Another factor to consider is that the CRISPR/Cas9 technique involves a laborious and drawn-out process of desirable transformed/mutated selection and regeneration. 3) The fact that some crops have a complicated genetic structure makes them difficult to study from a genomic perspective. Because of their quantitative nature, the genes relating to post-harvest quality traits in many crops are still mostly unstudied. The CRISPR/Cas9 technique has the ability to insert modifications crop genomes. The use of CRISPR/Cas has been constrained in many countries caused of the ambiguity of the current biosafety regimes (SDN-1, SDN-2, and SDN-3) (Eckerstorfer et al., 2019). Additionally, the proponents of the CRISPR/Cas9 tool are more concerned about this “overregulation” according to the GMO laws, not because there is not a specific or unique regulatory framework. Hence, fruits, vegetables, and ornamental crops must be allowed to use the CRISPR/Cas9 tool. Delivery protocols and easy transformation must be developed for the modification of the desired plant generation in the case of recalcitrant crops. Additionally, in the circumstances of the apple and grape, adopting DNA-free genome editing technology based on the RNP (ribonucleoprotein) complex yields a better outcome for successfully creating mutant lines (Malnoy et al., 2016a), and potatoes (Andersson et al., 2018) by biolistic gun and PEG-mediated transformation. Many nations have already accepted and developed genome editing technologies and successfully commercialized genetically altered crops. Most countries decide the regulatory status of genome-edited organisms on a case-by-case basis and can use the current regulatory framework for GMOs (Eckerstorfer et al., 2019). The worldwide regulatory environment for genome-edited species is far more heterogeneous than conventional GMOs. The regulatory triggers for biosafety laws vary across nations. Although International bodies, such as the OECD, fully acknowledge this fact yet are still striving to harmonize the regulatory control of biotechnology. It is difficult to see how this cooperation among experts and comprehension of all these technologies will successfully address this issue. Overall, the adoption and formulation of suitable regulations requirements to be increased effectively to apply the capability of this technology in the better crop improvement to require high-quality nutrient-rich food with accessibility to the continuously growing world population.

## 7 Concluding remarks

Traditional breeding merely depends on sufficient variability that exists among the plant populations that have made a great contribution to modern agricultural practices. However, variations generated either from spontaneous natural mutations or developed using chemical or physical mutagens, are random and in low-frequency events. Moreover, desired variations among the elite cultivars may not arise at the same time, and it is a delitory process. Conventional breeding fails to produce such traits in a limited period of time. In contrast, genome editing serves as a modern molecular biology tool that can produce error-free targeted modifications.

It is urgently necessary to provide this constantly expanding population with enough food that has better nutritional value on a worldwide scale. This could only be achieved in horticultural, and fruit crops with the acquisition of the latest genome editing technologies. However, there is a need for a transparent and smooth regulatory system, which can provide brooder applicability for the rapid delivery of genome-edited crops to the consumer market. In this present review, we have made an effort to deliver the great potentiality and versatility of genome editing to enhance the quality of traditional vegetables, fruits, and ornamental plants in a very concise way. In many underdeveloped countries, the significant causes of vegetable and fruit loss are because of inadequate infrastructure. In contrast, in developed countries, the losses occur at the consumer level. The use of convenient technology to control post-harvest yield loss is a demanding process. Genome editing is a promising method for rapid trait improvement, considering ornamental plants. But three possible drawbacks exist, which need to be addressed. Firstly, there is a need for suitable transformation methods to deliver genome editing reagents in the target tissue. Secondly, proper identification of genes related to a specific trait with agronomic importance is still required. Recent advancement in genome sequencing technology has made it easy to sequence many diploid ornamental plants, but it remains a challenge to identify genes specific to the particular trait of interest. In the case of polyploid flowers like *Chrysanthemums*, and roses, the WGS is not available. The proteome and transcriptome of these ornamental plants can pave the way forward for the identification of specific genes of interest at a particular time or environmental condition. Regardless of these limitations, genome editing serves as an extraordinary and outstanding approach to modifying horticultural crops for improving required traits and expanding the availability of ornamental plants for benefit of the society.
